# “Burrata di Andria” PGI Cheese: Physicochemical and Microbiological Features

**DOI:** 10.3390/foods9111694

**Published:** 2020-11-19

**Authors:** Alessandro Di Cerbo, Dino Miraglia, Leonardo Marino, Roberta Stocchi, Anna Rita Loschi, Stefano Fisichella, Natalina Cammertoni, Laura Menchetti, Silvana Farneti, David Ranucci, Raffaella Branciari, Stefano Rea

**Affiliations:** 1 School of Biosciences and Veterinary Medicine, University of Camerino, 62024 Matelica, Italy; roberta.stocchi@unicam.it (R.S.); annarita.loschi@unicam.it (A.R.L.); natalina.cammertoni@unicam.it (N.C.); stefano.rea@unicam.it (S.R.); 2 Experimental Zooprophylactic Institute of Umbria and Marche ‘T. Rosati’, 06126 Perugia, Italy; dino.miraglia@unipg.it (D.M.); s.farneti@izsum.it (S.F.); 3 Experimental Zooprophylactic Institute of Puglia and Basilicata, 71121 Foggia, Italy; leomaleo16@gmail.com; 4 Department of Veterinary Medicine, University of Perugia, 06126 Perugia, Italy; s.fisichella@izsum.it (S.F.); laura.menchetti@studenti.unipg.it (L.M.); david.ranucci@unipg.it (D.R.); raffaella.branciari@unipg.it (R.B.)

**Keywords:** Burrata di Andria, PGI, raw milk, pasteurized milk, physicochemical features, microbiological features

## Abstract

In the last century, the exponential increase of industrial food production led to the disappearance of “Italian traditional niche products”. However, national regulations allowed the preservation of several of these products, including the burrata cheese. Twenty-one samples from three different batches of “Burrata di Andria” Protected Geographical Indication (PGI) were purchased from dairy factories of the PGI consortium. Moisture value of PGI Burrata cheese was significantly higher than that before the PGI release. Moreover, a significantly lower NaCl value was detected in PGI raw milk Burrata cheeses with respect to non-PGI ones, while an opposite situation was detected in pasteurized milk Burrata cheeses. As for pH, in all PGI products lower values were observed with respect to non-PGI products, which resulted significant only in pasteurized ones. No *Salmonella* spp., *Listeria monocytogenes,* and *Bacillus cereus* were detected, while nine samples were positive for a nonpathogenic strain of *Yersinia enterocolitica.* Total viable count (TVC) and *Escherichia coli* resulted significantly lower in pasteurized than in raw milk PGI Burrata cheese samples. Although samples analyzed can be considered microbiologically safe, these were borderline and/or unsatisfactory for *E. Coli* and coagulase-positive staphylococci (CPS) according to process hygiene criteria established by European regulation. Therefore, different strategies should be adopted to improve products hygiene in the considered dairy factories.

## 1. Introduction

In the last century, the exponential increase of industrial food production led consumers to a wide choice of cheap products, thus paving the way for the disappearance of “ Italian traditional niche products” often characterized by poorly standardized working processes and a very restricted commercial network. Fortunately, specific EU and National regulations allowed the preservation of several of these products, including the burrata cheese, based on a 25-year historical tradition [1,2]. According to the Ministerial Decrees n. 170/98 and n. 350/99, the burrata cheese was officially recognized as an “Italian traditional agricultural product” of Puglia region by means of Ministerial Decree of 14/06/2002 [3]. In 2016, the “Burrata di Andria” cheese received the PGI (Protected Geographical Indication) through the Regulation (EU) 2016/2103 [4].

This kind of cheese stemmed from the idea to reuse the residues of stretched curd from mozzarella cheese-making process combined with whey cream and to enclose them in a stretched curd envelope. It has a weight and a diameter ranging from 100 to 500 g and from 7 to 12 cm, respectively, and is characterized by a smooth, shiny, milky white-colored surface without crust, with an outer overlapping sheet fibrous texture, which releases a milky liquid after light compression [5].

The internal texture is white or ivory-white, creamy, not spreadable, and of variable consistency depending on the quality and quantity of the cream used to prepare the filling [6]. The taste is typical of a fresh dairy product tending to acid, whereas the smell is typical of cream, fragrant and delicate, with a hint of slightly sour milk [5]. Once the shaping is completed, it is left to harden for 20–30 min in cold water [6].

The shelf-life ranges from 3 to 5 days, if handmade, due to poor microbiological characteristics [7,8,9], while the use of a blower machine during forming and filling phase can extend the shelf-life until 20 days [10]. Indeed, due to the presence of an adequate air filtering system the blower machine ensures a microbiologically safe compressed air insufflation to form the envelope.

Although the consumption is advisable within 24 h from the production to better appreciate the organoleptic features [5,6], it must be stored at 4 °C but, according to the tradition, should be consumed after bain-marie warming to achieve a greater creaminess and more intense flavor and taste [11]. It is generally manufactured using pasteurized cow’s milk, then mixed with the acidified serum and calf rennet to reach a pH around 6.1 to 6.2 [7,8].

Resmini et al. (1999) and Tantillo et al. (2007) firstly reported physicochemical features of different artisanal burrata cheeses, which slightly differed due to the lack of a standardized production process [6,12]. Conversely, Rea et al. compared the physicochemical and microbiological features of artisanal and industrial burrata cheeses reporting a strong variability between the samples achieved with the two processes [13].

The product shows a certain degree of variability among cheese factories, representing a possible index of craftsmanship, albeit within the established minimum criteria required by a PGI product.

In fact, a PGI product should ensure a certain degree of standardization for some basic features to fulfill procedural guidelines.

The aim of this study was to evaluate physicochemical and microbiological features of samples of Burrata di Andria belonging to the PGI consortium and to compare them with the data obtained in a previous study from Italian traditional Burrata cheeses manufactured with raw or pasteurized milk in dairy factories without the procedural guidelines of PGI and processed according to the same analytical procedures [13].

## 2. Materials and Methods

Twenty-one samples from three different batches of “Burrata di Andria” PGI, were purchased from seven different dairy factories belonging to the PGI consortium.

All factories were considered artisanal due to the manual execution of several cheese-making steps (“pasta filata” envelope and filling preparation and final product shaping) and the main factor to discriminate among products was the use of raw or pasteurized milk. Three out of seven dairy factories used raw milk (*n* = 9) while the remaining four used pasteurized milk (*n* = 12).

The Burrata cheeses were transported to the laboratory in refrigerated conditions at 4 °C and analyzed within 12 h after collection. Each sample was aseptically subdivided in two aliquots, one for physicochemical and one for microbiological analysis.

### 2.1. Physicochemical Analyses

Physicochemical analyses were performed in triplicate on each sample obtained from inner and outer layer of the cheese homogenized with a blender (Model HGB2WT, Waring Commercial, Torrington, CT, USA).

Water activity (*a*_w_) was evaluated on 2 g of Burrata cheese using a BTRS1 Rotronic hygroscope (PBI International, Milan, Italy). pH was assessed by means of a pH meter equipped with an insertion electrode (Crison pH25, Crison, Barcelona, Spain). Moisture (948.12)*,* ash (935.42), fat (995.19), and NaCl (935.43) were determined according to Association of Analytical Chemists (AOAC) methods [14].

Proteins were determined by Kjeldahl method (991.20) using a Tecator™ digestion system (2006 Digestor, FOSS, Denmark) and distillation unit UDK 139 Kjeldahl (VELP Scientifica, Usmate (MB), Italy) [15]. The principle of the method consists in a digestion of the cheese in H_2_SO_4_ using CuSO_4,_ 5H_2_O as catalyst, performed on the digestion system at 400 °C for about 2 h. Digested solution containing nitrogen converted into (NH_4_)_2_SO_4_ is added with 40% NaOH to release NH_3_, which is distilled, collected into an H_3_BO_3_ solution and titrated using 0,1M HCl.

Fats were determined with “Mojonnier” method followed by solvent evaporation by a rotary evaporator RE 100 (PT. Murni Dharma Karya Chemical and Laboratory Supply, Jakarta Timur, Indonesia) [16].

### 2.2. Microbiological Analyses

From each Burrata cheese, 25 g of product were aseptically collected, mixed with 225 mL of sterile buffered peptone water (Oxoid, Basingstoke, UK) and homogenized for 60 s at room temperature (24 °C) in a Stomacher 400 (Stomacher 400 circulator; Seward Ltd., Norfolk, UK). The homogenates were serially diluted in sterile peptone water and used for the following determinations: total viable count (TVC) on Plate Count Agar (Oxoid), aerobically incubated at 30 °C for 48 h [17]; *E. coli* count using EC X-GLUC Agar (Chromogenic *E. coli*, Biolife) aerobically incubated at 44 °C for 24 [18]; enumeration of coagulase-positive staphylococci (CPS) on Baird Parker agar (Oxoid) with RPF supplement (Oxoid) incubated at 37 °C for 48 h [19]; *Bacillus cereus* determination on Mannitol Egg Yolk Polymyxin Agar (M.Y.P. agar, Oxoid) added with Egg Yolk Emulsion (Oxoid) and Polymyxin B Supplement (Oxoid) incubated at 30 °C for 24–48 h [20]. Each sample was analyzed in duplicate and the results were reported as Log CFU (colony forming units)/g of burrata. The averages were calculated using the countable values.

The remaining homogenates were incubated at 37 °C for 18 h (pre-enrichment phase) to evaluate the presence of *Salmonella* spp., followed by an enrichment in Rappaport Vassiliadis Soy Broth (Biolife) at 42 °C for 24 h and inoculated on Chromogenic *Salmonella* Agar Base (Oxoid) at 37 °C for 24 h [13].

*Yersinia enterocolitica* was evaluated by suspending 10 g of sample in 90 mL of *Yersinia* Sorbitol Peptone Broth and Bile Salts (PSB) broth (Oxoid) incubated at 25 °C for 3 days. After a treatment with 0.5% KOH solution, 0.1 mL were plated on *Yersinia* Selective Agar Base (Oxoid) supplemented with *Yersinia* Selective Supplement (Oxoid) incubated at 30 °C for 24 h [13]. The typical colonies were than confirmed as *Y. enterocolitica* and assessed for the presence of the virulence *ail* gene by multiplex polymerase chain reaction end point according to Garzetti et al. (2014) [21].

The presence of *Listeria monocytogenes* was also tested. Twenty-five g of each burrata sample were mixed with 225 mL of Half Fraser Broth (Oxoid) and incubated at 30 °C for 24 h for the primary enrichment. Then, 10 mL of Fraser Broth (Oxoid) inoculated with 0.1 mL of pre-enrichment broth, were incubated at 37 °C for 24 h for the secondary enrichment, which were surface-plated on Agar Listeria according to Ottaviani and Agosti (ALOA) Agar (Biolife) subsequently incubated at 37 °C for 24–48 h.

### 2.3. Statistical Analysis

Data were analyzed using GraphPad Prism 8 software (GraphPad Software Inc., La Jolla, CA, USA). All data are presented as the means ± standard deviation (SD) and were first checked for normality using the D’Agostino–Pearson normality test. A two-sample unpaired Student’s *t*-test was applied to analyze the differences in moisture, fat, proteins, ash NaCl, a_w_, pH, TVC, *E. coli,* and CPS between raw and pasteurized milk Burrata cheeses achieved before [13] and after PGI release. A *p* < 0.05 was considered significant.

## 3. Results

Average values of physicochemical parameters of all Burrata cheeses analyzed in the present study are reported in Table 1. No significant differences were observed.

Physicochemical parameters of PGI Burrata cheeses from the seven dairy factories are reported in Table 2.

Among different dairy factories significant differences could be observed for moisture, fat, and proteins (*p* < 0.05), ash (*p* < 0.05, *p* < 0.001 and *p* < 0.01) and for a_w_ (*p* < 0.05 and *p* < 0.001). No significant differences were reported for NaCl and pH.

Moisture value of all samples was generally compliant with the range of 60–70% fixed by the procedural guidelines of PGI Burrata cheese with a mean value of 70.01 ± 2.75%.

The most important values able to influence microbial growth (NaCl, a_w_ and pH) from PGI Burrata cheeses detected in the present study were compared to those reported by Rea et al. (2016) in Burrata cheeses before the PGI release [13]. The comparison is reported in Table 3.

Significantly higher moisture percentages (*p* < 0.001) could be observed in raw and pasteurized milk PGI products with respect to those before the PGI release. As far as it concerns NaCl, a significantly lower value was detected in PGI raw milk Burrata cheeses with respect to non-PGI ones (*p* < 0.05), while an opposite situation was detected in Pasteurized milk Burrata cheeses (*p* < 0.001). As for a_w_, no differences were observed, although a significantly higher value (*p* < 0.001) resulted from statistical analysis in raw milk PGI Burrata cheeses with respect to non-PGI raw milk products, probably due to a limited sample number.

As for pH, in all PGI products lower values were observed with respect to non-PGI products, which resulted significant only for pasteurized ones (*p* < 0.05).

Microbiological results are shown in Table 4. TVC ranged between 4.24 Log CFU/g and 7.99 Log CFU/g, *E. coli* between < 2 Log CFU/g (not detectable load) and 5.43 Log CFU/g, and CPS count between < 2 Log CFU/g and 2.83 Log CFU/g. Seven (33.3%) and fifteen (71.4%) cheese samples were below the detectable load for *E. coli* and CPS, respectively. No *Salmonella* spp., *Listeria monocytogenes,* and *Bacillus cereus* were detected, while nine samples (42.9%) were positive for a nonpathogenic strain of *Yersinia enterocolitica.*

**Figure 1:**
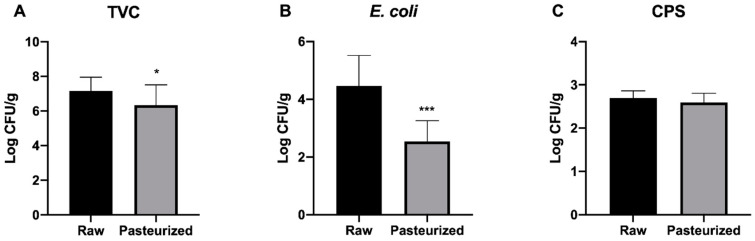
Comparison of TVC, *E. coli* and CPS counts between raw and pasteurized milk PGI Burrata cheeses. * *p* < 0.05, *** *p* < 0.001. (**A**) TVC, (**B**) *E. coli*, (**C**) CPS.

In Figure 1 TVC, *E. coli* and CPS values from raw (*n* = 9) and pasteurized (*n* = 12) milk PGI Burrata cheeses are compared.

TVC and *E. coli* resulted significantly (*p* < 0.05, *p* < 0.001) lower in pasteurized (6.34 ± 1.18 and 2.54 ± 0.72 Log CFU/g, respectively) than in raw (7.15 ± 0.80 and 4.47 ± 1.05 Log CFU/g, respectively) milk PGI Burrata cheeses samples. No significant differences were detected in CPS count.

**Figure 2:**
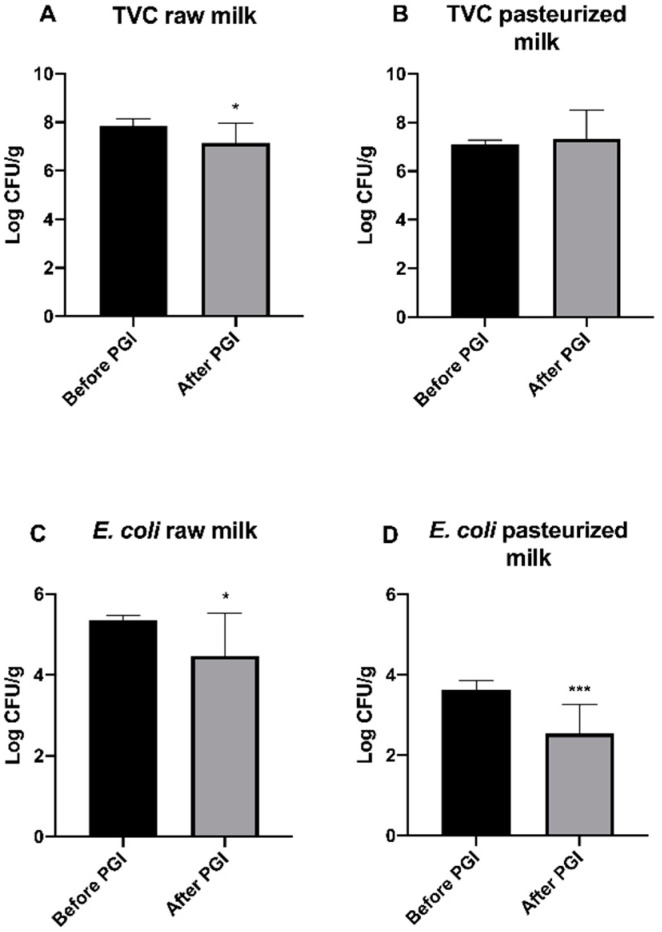
Comparison of TVC and *E. coli* in raw (**A** and **C**, respectively) and pasteurized (**B** and **D**, respectively) milk Burrata cheeses before (Rea et al., 2016) and after PGI release. * *p* < 0.05, *** *p* < 0.001.

The comparison of TVC count between raw milk Burrata cheeses before [13] and after PGI release revealed a significantly (*p* < 0.05) lower value in the latter (Figure 2A). No significant difference was observed between pasteurized milk samples (fig 2B). The concentration of *E. coli* resulted significantly lower in PGI than in non PGI samples (* *p* < 0.05 and ** *p* < 0.001, respectively) both from raw (4.47 ± 1.05 and 5.35 ± 0.11 Log CFU/g, respectively) and pasteurized (2.54 ± 0.72 and 3.62 ± 0.24 Log CFU/g, respectively) milk Burrata cheeses (Figure 2C,D). As for CPS (data not shown), values lower than 3 Log CFU/g were observed in all samples as also reported by Rea et al. (2016) [13].

## 4. Discussion

First of all, it must be emphasized that no differences in physicochemical features were detected between raw and pasteurized milk PGI Burrata cheeses. However, a remarkable variability can be observed for moisture, fat and NaCl among raw milk PGI Burrata cheeses, while a similar variability was detected for proteins, ash, and NaCl (at a lower extent) in the pasteurized milk products. In spite of such an overall degree of variability, the differences observed among dairy factories were significant only for a_w_ and ash. Such variability might be reasonably ascribed to different production technologies used in dairy factories. As for NaCl content, no direct correlation between its increase and the increase in moisture content was observed as conversely reported by Faccia et al. [22].

The comparison between the data of PGI Burrata cheeses and those of non-PGI reported by Rea et al. (2016) showed higher moisture percentages and lower pH in the formers.

The mean TVC (6.69 Log CFU/g) was lower compared to those reported by Rea et al. (2016) for Burrata cheeses made of raw milk (7.85 Log CFU/g; *p* < 0.01) while it did not differ from those made of pasteurized milk (7.11 Log CFU/g). Some authors retain that microbial counts higher than 6 Log CFU/g indicate poor hygienic quality [9]. In our study, fourteen Burrata cheeses (66.7%) exceeded this threshold, among these, nine (42.9%) had counts greater than 7 Log CFU/g. However, in dairy products TVC cannot be uniquely considered a process hygiene criterion as in other foods of animal origin, due to the inability to distinguish the contaminating microorganism from the lactic acid bacteria [13]. On the contrary, *E. coli* count provides useful indications on the hygiene level of the production process. This microorganism is considered an indicator of fecal contamination in food and its presence at high loads in pasteurized milk cheeses could be due to deficiencies in good manufacturing practices [23]. In this regard, a marginal limit (m) of 2 Log CFU/g and a maximum limit (M) of 3 Log CFU/g for *E. coli* are settled by Regulation (EC) n. 2073/2005 for pasteurized milk cheeses [24]. In the current work, 3 out of 12 pasteurized milk Burrata cheeses produced cheeses exceeding the acceptability limit of 2 Log CFU/g, while 1 the unsatisfactory level of 3 Log CFU/g (Data not shown).

In addition, four samples were also found to be positive for coagulase-positive staphylococci, with values ranging between 2 and 3 Log CFU/g. Although the counts were not worrisome for enterotoxin production (10^5^ CFU/g), the amount exceeded the limits indicated in Regulation (EC) 2073/2005 for pasteurized milk cheeses. In dairy products, raw milk and humans are considered the main sources of CPS contamination [25,26,27]; thus, in our study, their detection in pasteurized milk Burrata cheese can be linked to poor operators hygiene and incorrect handling [28].

However, these findings indicate better process hygiene than that previously observed by Rea et al. (2016) [13], who reported *E. coli* mean values of 3.62 Log CFU/g and 5.35 Log CFU/g for Burrata cheeses made of pasteurized and raw milk, respectively. In our study, stratifying the samples according to the heat treatment of the milk, *E. coli* mean values were 2.45 ± 0.70 (*p* < 0.01) and 4.47 ± 1.17 Log CFU/g for burrata cheeses made with pasteurized and unpasteurized milk, respectively. Moreover, Rea et al. (2016) found all artisanal samples positive for CPS, even though at low levels [13]. In another study evaluating different types of unripened raw milk cheese, Tirloni et al. (2014) [9] detected CPS in 100% of burrata cheeses (2–4 Log CFU/g) and very variable *E. coli* counts, with values between < 2 and 7.9 Log CFU/g. Instead Dambrosio et al. (2013) found good microbiological quality in Burrata cheeses, with an average *E. coli* values of 4.4 × 10^2^ CFU/g and CPS loads of 3 Log CFU/g only in 3.7% of samples. In another survey conducted on fifteen types of cheese, the same authors recorded a higher prevalence of CPS (18.2%) but lower mean *E. coli* values (3.9 × 10 CFU/g) in pasteurized milk Burrata samples [8].

As regard food safety criteria, no investigated pathogen was detected, although five samples produced with raw milk and four with pasteurized milk were found to be positive for *Y. enterocolitica* but as nonpathogenic strains. These results are in agreement with the data previously reported for the same type of cheese by Rea et al., (2016) [13]. No other evidences for *Y. enterocolitica* in Burrata cheeses are available in the literature. *Y. enterocolitica* is well known as a cause of yersiniosis in humans [29,30,31], but its real prevalence in food is unknown because no routine research is carried out for this pathogen [32].

## 5. Conclusions

Based on these results, the Burrata cheeses analyzed in this work can be considered microbiologically safe. However, according to process hygiene criteria established by European regulation, same samples were borderline and/or unsatisfactory for *E. Coli* and CPS, respectively, but still lower than five logs. Therefore, in the production plants considered different strategies should be adopted to improve products hygiene starting from milk quality, environmental and equipment sanitization, good manufacturing practices and staff training. Furthermore, due to mean physicochemical properties (NaCl < 0.45%, pH > 6 and a_w_ ≥ 97%), which do not limit the growth of pathogenic and spoilage microorganisms, proper management of the refrigeration temperature during storage and retail is strongly recommended for Burrata cheese [7].

## Figures and Tables

**Table 1:** Physicochemical parameters (mean ± SD) of total, raw and pasteurized milk Protected Geographical Indication (PGI) Burrata cheeses analyzed.

	Moisture (%)	Fat (%)	Proteins (%)	Ash (%)	NaCl (%)	a_w_	pH
Total (*n* = 21)	70.01 ± 2.75	17.89 ± 2.36	10.28 ± 2.11	1.81 ± 0.29	0.42 ± 0.21	0.97 ± 0.01	6.29 ± 0.20
Raw milk (*n* = 9)	69.39 ± 3.48	18.35 ± 2.33	10.50 ± 2.31	1.76 ± 0.29	0.45 ± 0.25	0.97 ± 0.004	6.21 ± 0.27
Pasteurized milk (*n* = 12)	70.48 ± 2.10	17.55 ± 2.43	10.12 ± 2.04	1.86 ± 0.30	0.39 ± 0.19	0.97 ± 0.008	6.35 ± 0.12

**Table 2:** Physicochemical parameters (mean ± SD) of PGI Burrata cheeses produced in different dairy factories.

	Cheese Factories						
	1 (Raw Milk)	2 (Pasteurized)	3 (Raw Milk)	4 (Pasteurized)	5 (Pasteurized)	6 (Pasteurized)	7 (Raw Milk)
Moisture (%)	66.47 ± 3.32 ^a^	69.04 ± 2.06 ^a,b^	70.44 ± 2.85 ^a,b^	71.26 ± 3.09 ^b^	70.01 ± 1.55 ^a,b^	71.56 ± 3.67 ^b^	71.30 ± 3.08 ^b^
Fat (%)	20.01 ± 1.82 ^a^	18.28 ± 2.41 ^a,b^	16.51 ± 2.67 ^a,b^	18.53 ± 2.79 ^a,b^	17.50 ± 2.98 ^a,b^	18.48 ± 2.56 ^a,b^	15.95 ± 2.86 ^b^
Proteins (%)	11.53 ± 1.61 ^a^	10.57 ± 1.62 ^a,b^	11.35 ± 2.53 ^a^	8.61 ± 2.50 ^a,b^	10.67 ± 1.78 ^a,b^	8.09 ± 2.88 ^b^	11.14 ± 1.16 ^a^
Ash (%)	1.99 ± 0.24 ^ac^	2.12 ± 0.16 ^c^	1.70 ± 0.35 ^a,b^	1.60 ± 0.06 ^b^	1.81 ± 0.33 ^a,b,c^	1.88 ± 0.16 ^b^	1.62 ± 0.29 ^b^
NaCl (%)	0.52 ± 0.29 ^a^	0.57 ± 0.2 ^a^	0.50 ± 0.29 ^a^	0.32 ± 0.10 ^a^	0.38 ± 0.21 ^a^	0.39 ± 0.007 ^a^	0.24 ± 0.07 ^a^
a_w_	0.97 ± 0.005 ^a,c^	0.97 ± 0.005 ^a,c^	0.97 ± 0.00 ^a^	0.98 ± 0.01 ^c^	0.97 ± 0.005 ^a,c^	0.96 ± 0.00 ^b^	0.97 ± 0.00 ^a^
pH	6.20 ± 0.04 ^a^	6.10 ± 0.41 ^a^	6.40 ± 0.04 ^a^	6.39 ± 0.22 ^a^	6.30 ± 0.02 ^a^	6.33 ± 0.06 ^a^	6.34 ±0.07 ^a^

Values followed by the same letter (a, b, c) in each row do not differ significantly (*p* < 0.05).

**Table 3:** Comparison between moisture, NaCl, a_w_ and pH (mean ± SD) values in raw (A) and pasteurized (B) milk Burrata cheeses before (Rea et al., 2016) and after PGI release. * *p* < 0.05, *** *p* < 0.001.

	Moisture (%)	NaCl (%)	a_w_	pH
**A**				
Non-PGI raw milk	59.46 ± 2.62	0.99 ± 0.56	0.96 ± 0.003	6.35 ± 0.14
PGI raw milk	69.39 ± 3.48 ***	0.45 ± 0.25 *	0.97 ± 0.001 ***	6.21 ± 0.27
**B**				
Non-PGI Pasteurized milk	62.59 ± 0.67	0.12 ± 0.01	0.97 ± 0.001	6.51 ± 0.15
PGI pasteurized milk	70.48 ± 2.10 ***	0.39 ± 0.19 ***	0.97 ± 0.01	6.35 ± 0.12 *

**Table 4:** Descriptive statistics (relative frequencies of positive cheese samples, maximum, minimum, mean, standard deviation, median) for total viable count (TVC), *E. coli,* and coagulase-positive staphylococci (CPS) (*n* = 21).

	Positive Samples (%)	Minimum * (Log CFU/g)	Maximum * (Log CFU/g)	Mean * (Log CFU/g)	SD * (Log CFU/g)	Median * (Log CFU/g)
TVC	100.0%	4.24	7.99	6.69	1.11	6.94
*E. coli*	66.7%	2.00	5.43	3.12	1.29	2.80
CPS	28.6%	2.00	2.83	2.45	0.35	2.48

* calculated using the countable values.
